# Long-term monitoring of the seasonal density of questing ixodid ticks in Vienna (Austria): setup and first results

**DOI:** 10.1007/s10493-020-00511-4

**Published:** 2020-06-18

**Authors:** Janna R. Vogelgesang, Melanie Walter, Olaf Kahl, Franz Rubel, Katharina Brugger

**Affiliations:** 1 Unit for Veterinary Public Health and Epidemiology, University of Veterinary Medicine Vienna, Veterinärplatz 1, 1210 Vienna, Austria; 2 tick-radar GmbH, 10555 Berlin, Germany

**Keywords:** *Ixodes ricinus*, *Haemaphysalis concinna*, *Dermacentor reticulatus*, Flagging, Dragging, Urban habitats, Monitoring

## Abstract

The first long-term monitoring to document both activity and density of questing ixodid ticks in Vienna, Austria, is introduced. It was started in 2017 and is planned to run over decades. Such long-term monitorings are needed to quantify possible effects of climate change or to develop tick density forecast models. The monthly questing tick density at three sites has been observed by using a standardized sampling method by dragging an area of $100\,\hbox {m}^2$ at each occasion. Popular recreational areas were chosen as study sites. These are the Prater public park, the wooded Kahlenberg, and a wildlife garden in Klosterneuburg. First results show a 3-year time series of nymphs and adults of the *Ixodes ricinus* species complex and *Haemaphysalis concinna* for the period 2017–2019. Whereas questing nymphs of the *I. ricinus* species complex were collected from February to November, *H. concinna* nymphs were only dragged from May to October. The peak of nymphal activity of the *I. ricinus* species complex was in May, that of *H. concinna* in August. In addition, a brief overview is given about ticks and tick-borne pathogens occurring in urban and suburban areas of Vienna.

## Introduction

Ixodid ticks (Acari: Ixodidae) play an important role in public and veterinary health as they are vectors of pathogens causing diseases such as tick-borne encephalitis (TBE), Lyme borreliosis (LB), or babesiosis in both humans and animals (Kahl 2018). Although there is a trend towards urbanization, urban and suburban green areas still provide attractive habitats for ticks (Dautel and Kahl 1999). There are two major requirements for establishing and preserving independent tick populations in urban areas: suitable environmental conditions, particularly microclimate (temperature, humidity) and the availability of appropriate hosts (Uspensky 2014b). The latter includes small mammals such as rodents or birds, medium-sized hosts such as hedgehogs or squirrels, and large hosts such as deer, wild boar, or foxes (Uspensky 2014a). The metropolitan area of Vienna (Austria) provides a variety of green areas suitable for ticks: Vienna Woods, the riparian woodland along the Danube, public parks (e.g., Prater, Lainzer Tiergarten, Schwarzenbergpark), natural cemeteries (e.g., Vienna Central Cemetery), and private gardens just to mention a few (Radda et al. 1986; Blaschitz et al. 2008; Wijnveld et al. 2016).

In the urban and suburban areas of Vienna, *Ixodes ricinus* is the most common tick species, followed by *Dermacentor reticulatus* and *Haemaphysalis concinna*, with *I. ricinus* being the most important vector of pathogens (Radda et al. 1986). The recently described species *Ixodes inopinatus* could also be a vector of pathogens (Estrada-Peña et al. 2014; Chitimia-Dobler et al. 2018). Although ticks and the occurrence of tick-borne pathogens have been investigated in numerous studies in Vienna (Blaschitz et al. 2008; Leschnik et al. 2012; Wijnveld et al. 2016; Schötta et al. 2017; Weiler et al. 2017), a long-term monitoring of the questing tick density is still lacking. However, such a long-term monitoring over several decades is needed to quantify possible effects of climate change or to develop tick density forecast models. Tick occurrence and density should be monitored to assess the risk of tick bites in humans and animals. In particular, an estimation of the questing tick density by using a standardized sampling method is essential. Time series of questing tick densities were recently used to compile tick density maps for Germany (Boehnke et al. 2015; Brugger et al. 2016), to investigate seasonal cycles of the tick density (Brugger et al. 2017), and to develop models to forecast the next season’s tick densities (Brugger et al. 2018).

Several methods are available for monitoring both activity and density of exophilic ticks: flagging or dragging of questing ticks (Estrada-Peña et al. 2013), collecting ticks from hosts, e.g., small mammals (Pfäffle et al. 2011) or dogs (Duscher et al. 2013), capturing ticks with $\hbox {CO}_2$-baited sticky traps (Gray 1985), or monitoring questing ticks on field plots (Dautel et al. 2008). All methods and their variations have advantages and disadvantages as discussed by Dobson (2013) or Mays et al. (2016). Nevertheless, flagging or dragging is a reliable method, which is rather easy to implement and gives good insights into tick population dynamics. The simple method of dragging a white blanket over the vegetation has been varied by using a sledge (Cornet et al. 1984) or sweep (Carroll and Schmidtmann 1992) instead of a pole, or combined with carbon dioxide release (Gherman et al. 2012).

The aim of this work was to document the setup of the first long-term questing tick density monitoring at three typical recreation sites in urban and suburban areas of Vienna. Further, the results of the first 3 years of that ongoing study along with tick-borne pathogens detected in Vienna are discussed.

## Materials and methods

### Study areas

**Fig. 1:**
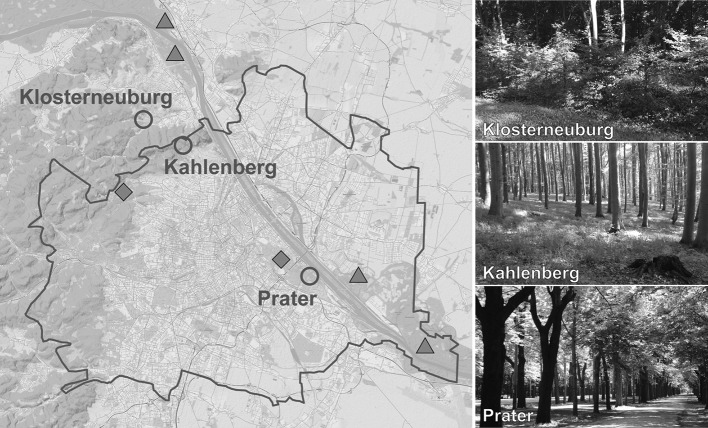
The three long-term monitoring sites Klosterneuburg, Kahlenberg, and Prater in urban and suburban areas of Vienna (red circles). Additionally, sites where *Ixodes ricinus*, *Haemaphysalis concinna*, and *Dermacentor reticulatus* were detected in previous studies are marked with grey triangles and those with only *I. ricinus* with grey diamonds (Radda et al. 1986; Blaschitz et al. 2008; Leschnik et al. 2012; Wijnveld et al. 2016; Schötta et al. 2017; Weiler et al. 2017)

**Fig. 2:**
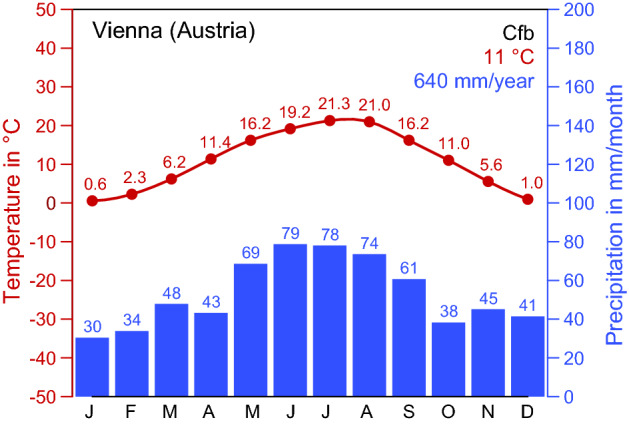
Climate diagram for Vienna $(16.4^\circ \,\hbox {E}/48.2^\circ \,\hbox {N})$ for the period 1986–2010

To determine the seasonal activity and density of questing ticks in urban and suburban areas of Vienna, a monitoring program in three popular recreation areas, Klosterneuburg, Kahlenberg, and Prater, was started in spring 2017 (Table 1, Fig. 1). With a population of about 1.9 million and an area of nearly $415\, \hbox {km}^2$, Vienna is the largest city of Austria located in Central Europe. Approximately 45% $(190~\hbox {km}^2)$ of the total city area are green areas consisting of 44% forest, 31% agriculture areas, 12% grasslands, and 13% parks, sport grounds, and cemeteries (Municipal Department 23 – Economic Affairs, Labour and Statistics, City of Vienna 2018). The suburbs of Vienna are characterised by villages and small municipalities with detached houses and gardens, agricultural areas such as the Marchfeld plain, and nearby recreation areas such as the Danube Floodplain National Park and the Vienna Woods. The climate in the urban and suburban areas of Vienna is warm temperate with rain in all seasons and warm summers (Fig. 2). Corresponding to the widely used Köppen-Geiger climate classification it is referred to as Cfb climate (Rubel et al. 2017).

**Table 1:** Geographic locations of the three monitoring sites and the associated Corine land cover class (European Environment Agency 2016)

Site	Longitude (° E)	Latitude (° N)	Altitude (m)	Land class
Klosterneuburg	16.29174	48.28761	265	Agricultural area
Kahlenberg	16.32625	48.27703	459	Broad-leaved forest
Prater	16.44240	48.19426	164	Urban area

The three monitoring sites are located in typical recreation areas in Vienna and a nearby municipality. One site is located in the Prater, a large public park close to the city centre of Vienna with a natural riparian zone and a wide variety of open areas. This former royal hunting ground is one of the largest recreation areas in Vienna, which is much-frequented by families, walkers, joggers, and dog and horse owners. The Prater is an extended area of meadows, broad-leaved forest with dense undergrowth, and former Danube arms. Regularly mowed lawns, meadows, and forest are crossed by the more than 4 km long Prater main avenue and various cycle paths, riding trails, and hiking trails. According to the tree cadastre of the Vienna city administration (Municipal Department 42 – Parks and Gardens, City of Vienna 2019) the dominant tree species are horse chestnut (*Aesculus hippocastanum*), black poplar (*Populus nigra*), and common ash (*Fraxinus excelsior*). Typical shrubs are elderberry (*Sambucus nigra*), common hazel (*Corylus avellana*), and guelder rose (*Viburnum opulus*). The Prater gives habitat for a variety of wild animals including small rodents, hedgehogs (*Erinaceus europaeus*), red squirrels (*Sciurus vulgaris*), and birds, and game such as roe deer (*Capreolus capreolus*), red fox (*Vulpes vulpes*), and wild boar (*Sus scrofa*).

The site Kahlenberg is a mountain located in the Vienna Woods, a largely protected green belt with forest and meadows at the western border of Vienna. It is a popular destination and viewpoint not only for tourists but also for runners, hikers, and dog owners. The Viennese longest road Höhenstraße meanders with many serpentines through the forest up the top of Kahlenberg, almost 300 m above downtown Vienna. The Vienna Woods are the largest contiguous broad-leaved forest in Central Europe, dominated by European beech (*Fagus sylvatica*), European hornbeam (*Carpinus betulus*), and European oak (*Quercus robur*). In spring, the herb layer is covered by wood garlic (*Allium ursinum*) and sweet woodruff (*Galium odoratum*). The forest provides habitat for several wild animals and is crossed by game paths and mud holes of wild boar.

The third site is located in and around a wildlife garden in Klosterneuburg, a municipality northwest of Vienna in the federal state Lower Austria. This site is embedded in a typical suburb structure with detached houses with gardens, adjacent broad-leaved forest, and agricultural areas (mainly vineyards). The garden has an old stock of broad-leaved and coniferous trees with mainly large-leaf linden (*Tilia platyphyllos*), field maple (*Acer campestre*), and Scots pine (*Pinus sylvestris*), as well as young trees and partly dense shrubs along the fences. The meadow is mowed several times a year, and the garden is frequented by wild animals and crossed by game paths due to the proximity of the forest and a holey fence.

### Tick sampling

At each of the three sites questing ticks were collected on a monthly schedule from February to November (i.e., 10 excursions per year). Relevant studies in Central Europe—e.g., by Schulz et al. (2014)—have demonstrated that questing *I. ricinus* occur very rarely in December or January. The monitoring began at the site Klosterneuburg in April 2017 and at the sites Prater and Kahlenberg in May 2017. Until now time series for the period 2017–2019 were compiled.

For tick dragging a 1x1-m white flannel cloth (soft cotton) was attached to a fiberglass flagpole and dragged over the leaf litter, woodland scrub, or low vegetation to drag an area of $100\,\hbox {m}^2$ on each occasion. The excursions were conducted on dry days (i.e., no rain) without strong wind to avoid wet substrate, which would affect dragging. Within an area of at least 1–2 ha the dragging transects were randomly selected at each excursion. This approach prevented excessive removal of ticks in a small area. After each 10-m transect the flannel was scanned and ticks were collected and transferred with a brush or tweezers into a vial for transportation to the laboratory. After the last transect, both sides of the flag were examined for ticks. Collected larvae were not recorded as they usually occur in clusters. With the dragging method questing ticks, i.e., lying in ambush for a passing host, are collected. In Austria, the exophilic ticks of the genus *Ixodes*, *Haemaphysalis*, and *Dermacentor* are present, but *I. ricinus* is considered the most common (Radda et al. 1986).

### Tick determination

Tick species and stages (nymphs, adult females, adult males) were morphologically determined under a stereo microscope (Leica S8APO) using the identification keys from Hillyard (1996) and Estrada-Peña et al. (2017). To distinguish between the very similar species *I. ricinus* and *I. inopinatus* the keys from Estrada-Peña et al. (2014) and Chitimia-Dobler et al. (2018) were used. To compare the results of this study with others published before 2014, these two species were combined under the term *I. ricinus* species complex. Finally, all ticks of each species as well as stages were counted and documented as monthly questing tick densities, i.e., numbers per $100\,\hbox {m}^2$.

## Results

**Fig. 3:**
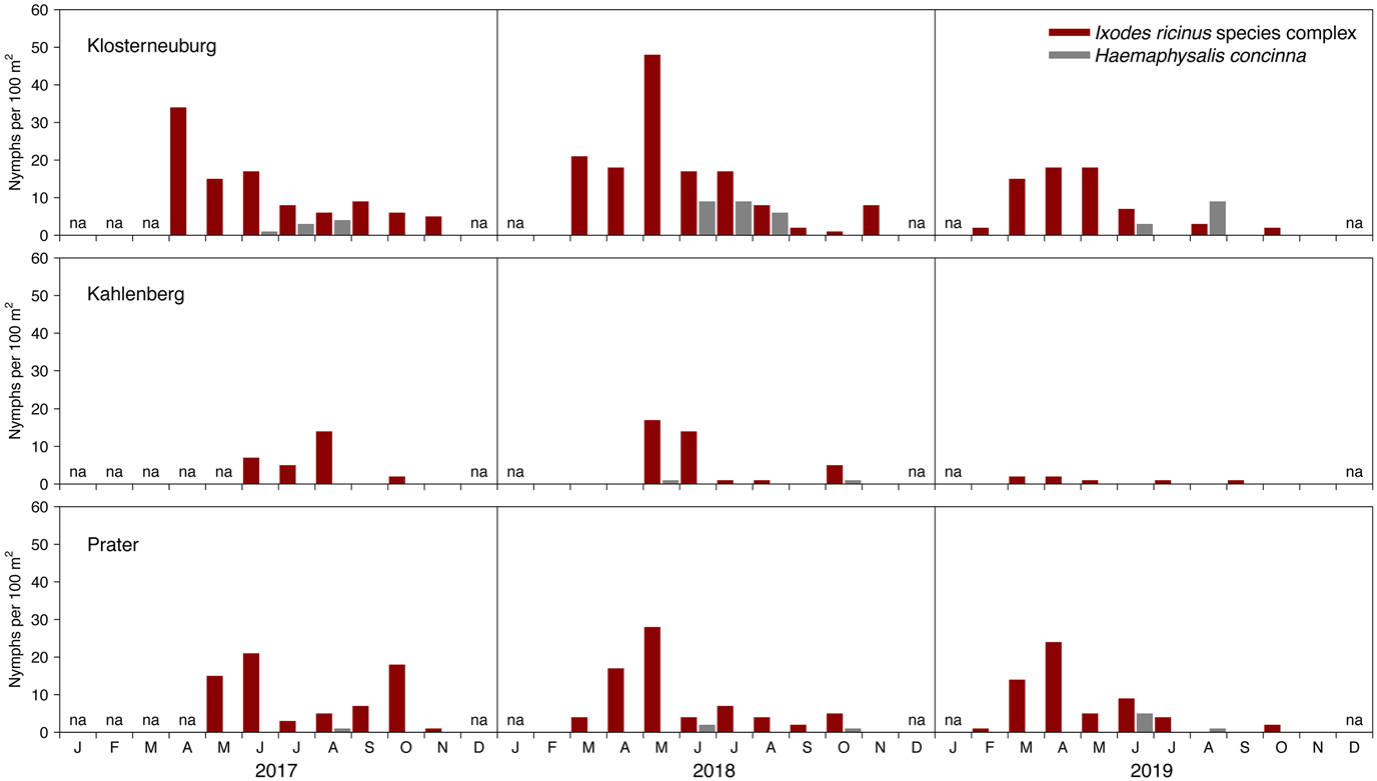
Monthly densities of questing nymphal ticks of the *Ixodes ricinus* species complex and *Haemaphysalis concinna* (unit: nymphs per $100\,\hbox {m}^2$) at the monitoring sites Klosterneuburg, Kahlenberg, and Prater in urban and suburban areas of Vienna (Austria) for the period 2017–2019. Months without excursion are indicated with na

**Fig. 4:**
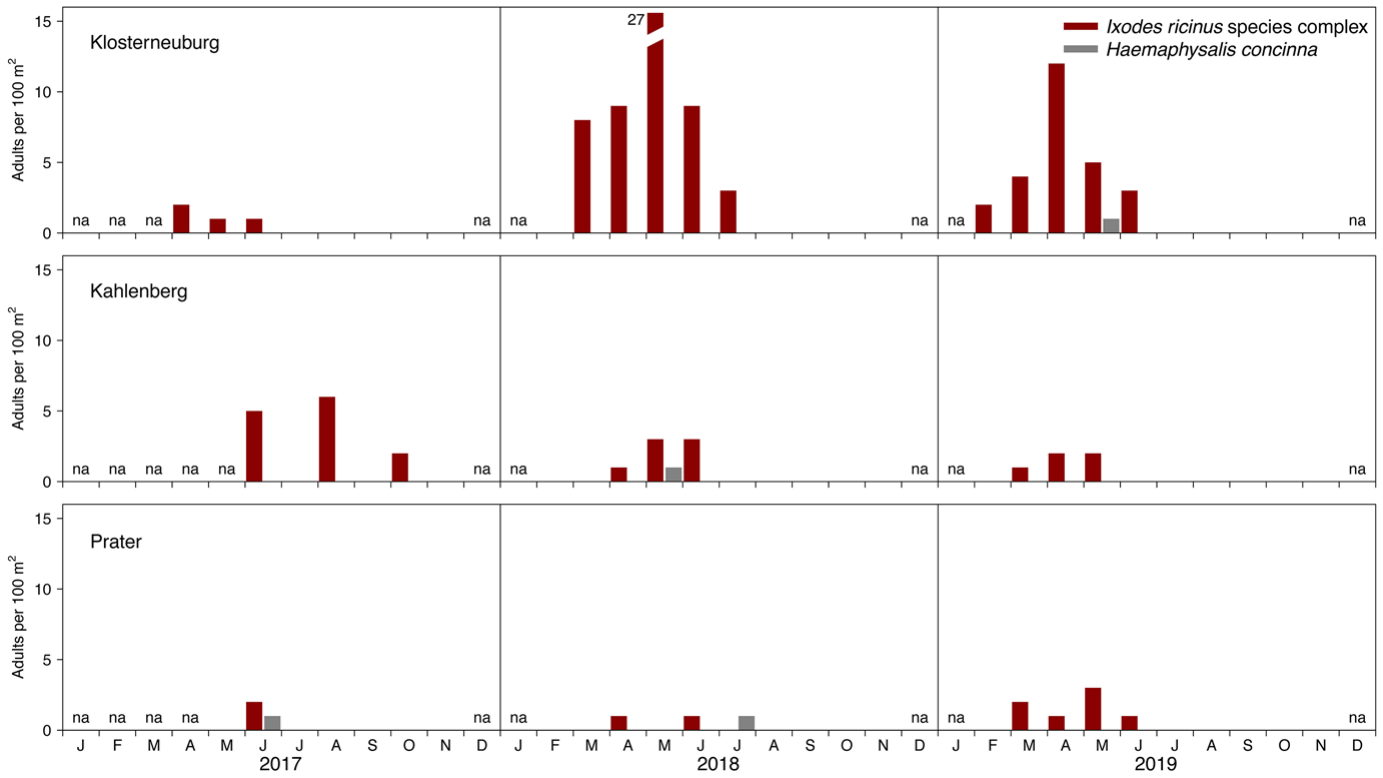
Monthly densities of questing adult ticks of the *Ixodes ricinus* species complex and *Haemaphysalis concinna* (unit: adults per $100\,\hbox {m}^2$) at the monitoring sites Klosterneuburg, Kahlenberg, and Prater in urban and suburban areas of Vienna (Austria) for the period 2017–2019. Months without excursion are indicated with na

Within the first three monitoring years (2017–2019) a total of 634 nymphal and 126 adult hard ticks were collected and identified to species and stage (Figs. 3 and 4). The highest number of nymphal ticks (55.1%, n = 349) were collected at the site Klosterneuburg followed by the site Prater (33.1%, n = 210), and the site Kahlenberg (11.8%, n = 75). The ranking for adult ticks is Klosterneuburg (69.0%, n = 87), Kahlenberg (20.6%, n = 26), and Prater (10.3%, n = 13).

The majority of the collected nymphal ticks belonged to the *I. ricinus* species complex (91.2%, n = 578) but also few *H. concinna* nymphs (8.8%, n = 56) were collected, especially during the summer months. Similar observations were made for adult ticks (*I. ricinus*: 96.8%, n = 122, *H. concinna*: 3.2%, n = 4). Pooled over all three sites and years, the share of *I. ricinus* vs. *I. inopinatus* was 93.8 vs. 6.2% for nymphs, and 98.4 vs. 1.6% for adults.

The 3-year time series of questing nymphal tick densities observed at the three sites are shown in Fig. 3. For both ticks of the *I. ricinus* species complex and *H. concinna* little to no activity was observed in the autumn. No excursion was conducted in December and January. The majority of nymphal ticks belonging to the *I. ricinus* species complex were collected from April to June (61.8%). In the extraordinary tick year 2018 (Brugger et al. 2018), almost 20% more nymphal ticks and 12× more adults were collected at the site Klosterneuburg than in the same period of the previous year.

**Fig. 5:**
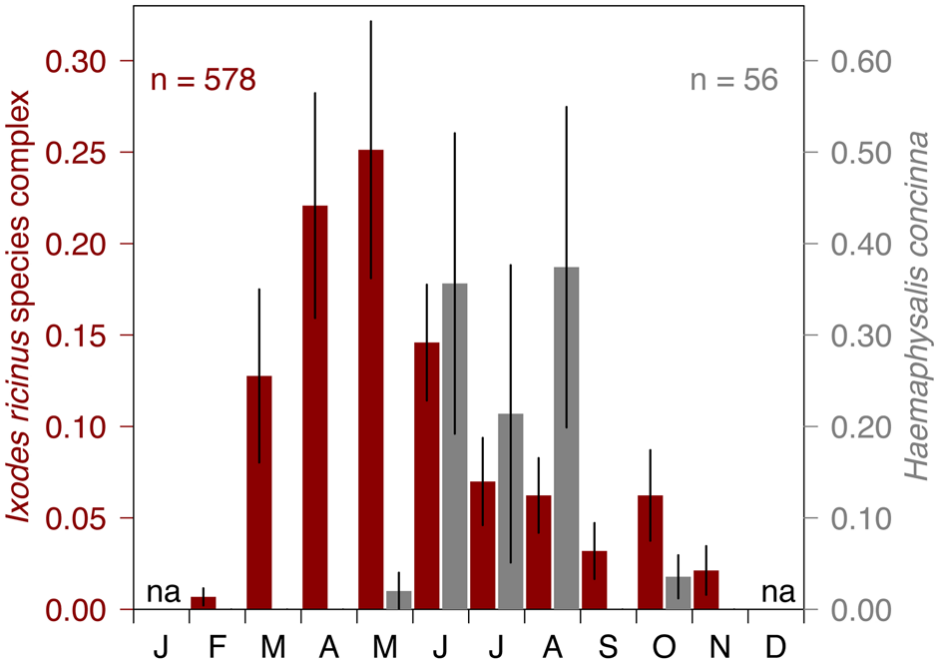
Fraction of the mean monthly density of questing nymphal ticks in Vienna (Austria). The standard error is given as measure of dispersion. Period: 2017–2019. Months without excursion are indicated with na

As shown in Fig. 5, nymphs of the *I. ricinus* species complex were questing nearly year round from February to November with a major peak in April and May and a second smaller peak in October. Contrary, *H. concinna* nymphs were only dragged from May to October with a peak in August. The standard error of the mean is given as measures of dispersion. A high variability of the questing tick density in connection with the still small sampling size of the 3-year study period leads to relatively large standard errors.

## Discussion

In the present study, ticks of the *I. ricinus* species complex were by far the most abundant. The bimodal seasonal activity of the nymphs with a major peak in April and May and a second smaller peak in October corresponds to other observations in urban and suburban areas in countries adjacent to Austria. These studies were conducted for example in the German cities Munich, Regensburg, Augsburg, Hannover and Berlin (Schorn et al. 2011; Hauck et al. 2020; Vollack et al. 2017), in the Czech cities Prague and Brno (Daniel et al. 2015; Žákovská et al. 2013), in the Slovakian cities Bratislava, Košice,and Bardejov (Kazimírová et al. 2016; Pangrácová et al. 2013), in the Croatian city Zagreb (Vucelja et al. 2020), and the suburbs in the southeast of Vienna (Duscher et al. 2013). In previous studies *Anaplasma phagocytophilum*, *Babesia* spp., *Bartonella* spp., *Borrelia* spp., and *Rickettsia* spp. were detected in nymphal and adult *I. ricinus* collected in Vienna (Table 2). Further, the annually reported human cases suggest an ongoing TBE virus circulation in Vienna (Heinz et al. 2015).

**Table 2:** Pathogens detected in different tick species and stages (nymph/adult) collected in urban and suburban areas of Vienna (Austria) in the last 40 years

Pathogens	*Ixodes ricinus*	*Haemaphysalis concinna*	*Dermacentor reticulatus*	Reference
*Anaplasma phagocytophilum*	Ny/Ad	Ny/Ad		Leschnik et al. (2012), Schötta et al. (2017)
*Babesia* spp.	Ny/Ad			Blaschitz et al. (2008), Schötta et al. (2017)
*Bartonella henselae*	Ny/Ad			Müller et al. (2016)
*Bartonella doshiae*	Ny/Ad			Müller et al. (2016)
*Borrelia* spp.	Ny/Ad	Ny/–		Radda et al. (1986), Leschnik et al. (2012) Schötta et al. (2017)
*Neoehrlichia mikurensis*	Ny/Ad			Schötta et al. (2017)
*Rickettsia* spp.	Ny/Ad	Ny/Ad	Ny/Ad	Rehácek et al. (1997), Leschnik et al. (2012), Schötta et al. (2017)
*Rickettsia raoultii*			–/Ad	Duscher et al. (2016), Wijnveld et al. (2016)

The second most common tick species collected in this study was *H. concinna*, a proven vector of a variety of pathogens (Rubel et al. 2018). *Anaplasma phagocytophilum*, *Borrelia* spp., and *Rickettsia* spp. were already detected in nymphal and adult ticks collected in Vienna and surroundings (Table 2). This tick species was collected mainly in the summer months and this corresponds well to the on-host activity pattern as observed on naturally infested dogs from Eastern Austria (Duscher et al. 2013).

Another tick species already detected in Vienna, but not within this study, is the meadow tick *D. reticulatus*. This tick is more likely to be found in the riparian woodland along the Danube (Fig. 1). Of the many pathogens this tick species can transmit (Rubel et al. 2016) only *Rickettsia* spp. have been detected in Vienna so far (Table 2).

The three tick species mentioned above may attack and feed on humans and their companion animals more or less frequently. Unfortunately, no study on the frequency of tick bites on humans was carried out in Vienna yet. The *Borrelia burgdorferi* sensu lato prevalence of 26.7% among *I. ricinus* ticks (Schötta et al. 2017) and annual notified human TBE cases (2% of all Austrian TBE cases) emphasize the medical significance of ticks and tick-borne diseases in urban and suburban areas in Vienna.

In the interests of completeness, also the European pigeon tick *Argas reflexus*, the hedgehog tick *I. hexagonus*, and the exotic brown dog tick *Rhipicephalus sanguineus* are increasingly common in the urban environment (Uspensky 2014b) and were occasionally detected in Vienna in the past decades (e.g., Strouhal 1947; Duscher et al. 2013; Prosl and Kutzer 1986).

Studies on ticks and tick-borne pathogens in urban and suburban areas have been systematically conducted since the 1980s. To date, the majority of the studies have focused on the tick-borne pathogen prevalence in ticks and less on tick density and activity in urban environments (Akimov and Nebogatkin 2016). Two studies were conducted in urban and suburban areas in two cities not far away from Vienna: a six-year time series in Prague, approximately 250 km away from Vienna (Daniel et al. 2015) and a three-year time series in Bratislava, 55 km away from Vienna (Kazimírová et al. 2016). These time series were used to identify seasonal beginning and cessation of questing activity and to predict the risk of humans of being bitten by ticks under certain weather conditions.

Here we present the implementation and the first results of the long-term monitoring of ticks in typical recreation areas in urban and suburban areas of Vienna. A successful long-term monitoring requires a cost-effective design for implementing and operational running over years or decades (Caughlan and Oakley 2001). Although monthly monitoring of three sites by dragging an area of $100\,\hbox {m}^2$ might be a limiting approach, it can be continuously conducted without major external funding for decades. For many species a decade is the minimum time required to detect population trends (White 2018). Long time-series are needed to quantify possible effects of climate change or to develop tick density forecast models. The latter can be used to define seasonal risk periods within which humans and their companion animals can acquire a tick bite and so may become infected with a tick-borne pathogen.
